# Association between Interpersonal Trust, Reciprocity, and Depression in South Korea: A Prospective Analysis

**DOI:** 10.1371/journal.pone.0030602

**Published:** 2012-01-18

**Authors:** Seung-Sup Kim, Yeonseung Chung, Melissa J. Perry, Ichiro Kawachi, S. V. Subramanian

**Affiliations:** 1 Department of Environmental and Occupational Health, The George Washington University School of Public Health and Health Services, Washington, D. C., United States of America; 2 Department of Mathematical Sciences, Korea Advanced Institute of Science and Technology, Daejeon, Republic of Korea; 3 Department of Society, Human Development, and Health, Harvard School of Public Health, Boston, Massachusetts, United States of America; Wayne State University, United States of America

## Abstract

**Background:**

A growing body of empirical evidence indicates that low-level social capital is related to poor mental health outcomes. However, the prospective association between social capital and depression remains unclear, and no published studies have investigated the association with longitudinal data in East-Asian countries.

**Methods:**

We analyzed data from the ongoing Korean Welfare Panel Study to prospectively investigate association between social capital and depression. Social capital was measured at the individual level by two items specific to interpersonal trust and reciprocity. Depression was annually assessed as a dichotomous variable using the Center for Epidemiologic Studies Depression Scale. After excluding participants who had depression in 2006, logistic regression models were applied to estimate the association between each social capital indicator and new-onset depression developed in 2007 or long-term depression in both 2007 and 2008. We also examined the association in a subpopulation restricted to healthy participants after excluding individuals with any pre-existing disability, chronic disease, or poor self-rated health condition.

**Results:**

Compared to the high interpersonal trust group, the odds ratios of developing new-onset and long-term depression among the low interpersonal trust group were 1.22 (95% CI: 1.08∼1.38) and 1.23 (95% CI: 1.03∼1.50), respectively, and increased to 1.32 (95% CI: 1.10∼1.57) and 1.47 (95% CI: 1.05∼2.08) in the subpopulation analyses restricted to healthy individuals. Although the low and intermediate reciprocity group also had significantly higher odds of developing new-onset depression compared to the high reciprocity group, the effects were attenuated and statistically non-significant in the subpopulation analyses.

**Conclusion:**

Low interpersonal trust appears to be an independent risk factor for new-onset and long-term depression in South Korea.

## Introduction

A growing body of empirical evidence demonstrates that low-level social capital is related to poor mental health outcomes such as depression [1], [2], [3], [4], [5], [6], psychosis [4], and suicide [7], [8]. These relationships were shown in different age groups: children [1], adolescents [3], elderly [2], and adults [4], [5], [6], [9], [10]. Most previous studies used a cross-sectional design, which cannot provide information about temporal order between social capital and mental health outcomes [1], [2], [3], [7], [8], [9], [11], [12], [13], [14], [15], [16]. This lack of evidence is critical considering that poor social capital could results in mental illness [17].

Several longitudinal studies were conducted to examine the impact of social capital on depression in Nordic countries and the U.S. For example, using a cohort of public sector employees in Finland, prospective studies showed that poor social capital at the workplace is an independent risk factor for new-onset depression [5], [18]. However, the prospective association between social capital and depression is still under debate. One Swedish study with 4.5 million participants showed the relationship between linking social capital, which connects people across different social divisions, and hospitalization due to depression, but the association became non-significant after an adjustment for potential confounders [4]. One study in the U.S. showed that a high level of individual trust in neighbors has a protective effect on the development of major depression, but the association was attenuated and became non-significant when the study population was restricted to non-depressed subjects at baseline [6]. Similarly, the impact of social capital on mental health is not clear in Asian countries including South Korea because most of the studies conducted with Asian populations focused on cross-sectional association [11], [12], [13], [14], [15], [16].

In the present study, we examined a prospective association between individual-level social capital and new-onset and long-term depression using nationally representative data from South Korea collected annually from 2006 to 2008. We constructed our study population using participants who were not depressed at baseline (2006) and followed them for two consecutive years. We investigated the association between social capital and depression after adjusting for potential confounders measured at baseline.

## Methods

Data were obtained from the Korean Welfare Panel Study (KOWEPS), an ongoing, annual, longitudinal study of a representative sample of 18,856 participants from 7,072 households at baseline in South Korea [www.koweps.re.kr] [18]. The KOWEPS was launched in 2006 by the Korean Institute of Social and Health Affairs in conjunction with the Social Welfare Research Institute of Seoul National University. The 1^st^ wave survey was conducted between November and December 2006, and the 2^nd^ and 3^rd^ wave surveys were conducted between April and July in 2007 and 2008. Data were collected through in-person interviews conducted by trained personnel. Data from the 1^st^ wave through the 3^rd^ wave (2006–2008) have been publicly released [www.koweps.re.kr]; the follow-up rate was 84% for the 3^rd^ wave survey [19].

### Individual-level social capital and depression

Two items specific to individual-level social capital were measured at baseline (2006) through a questionnaire. First, interpersonal trust was assessed through the question, “Do you think that most people are reliable?”; participants could answer “Most of them are reliable” (coded as high interpersonal trust), “We should be very careful” (coded as low interpersonal trust), or “I do not know.” Second, reciprocity was measured using the question, “Are you willing to help your neighbor who urgently needs your help (e.g., blood donation)?”; participants could answer on an ordinal scale with five levels (1: strongly no, 2: no, 3: neither no nor yes, 4: yes, 5: strongly yes). The score was categorized into three levels (low: 1 & 2, intermediate: 3, high: 4 & 5) for the analysis. These single item measurements of interpersonal trust and reciprocity were adopted in the previous studies [6], [20], [21].

The depression score was measured annually from 2006 to 2008 using an 11-question version of the Centers for Epidemiologic Studies Depression (CES-D) scale questionnaire [22], [23]. Several validation studies showed that the CES-D score has a reasonable psychometric property in East-Asian countries including South Korea [24], [25], [26], [27]. Because a dichotomizing cut-off score in the standard 20-question version CES-D scale is 16 for depressive symptoms, a score of 9 was used as a cutoff in our analysis for the 11-question version CES-D, in which the summation ranged from 0 to 33 [23]. Although the cut-off was created to screen for depressive symptoms, a participant with a score of 9 or higher was considered as depressed in our research. Depression in 2007 was termed “new-onset” depression, while depression in both 2007 and 2008 was termed “long-term” depression in this paper.

### Potential confounders

Potential confounders measured at baseline include gender, age, education, marital status, income, employment status, and health condition. Education was coded into four dummy variable categories: junior high or less, high school graduate, college graduate, and university graduate or more. Marital status was divided into currently married, never married, and previously married. To calculate an equivalized household income, the sum of household income from all sources including earning, interest, rent, and dividends, was divided by the square root of the number of household members. A categorical variable for income was then generated with four levels using the quartiles of the calculated equivalized income. Employment status was classified into seven categories: precarious worker, non-precarious worker, employer, self-employed worker, full-time student, unpaid family worker, and unemployed, including housewives. Waged workers were divided into precarious workers and non-precarious workers. Precarious workers were defined as waged workers under temporary/daily employment or part-time employment. Workers not fitting the precarious employment category were defined as non-precarious workers. Determination of precarious versus non-precarious was guided by previous study results indicating that, in South Korea, precarious workers are the disadvantaged group compared to non-precarious workers in terms of wage, social benefit, labor union, and health status [28], [29]. Participants who were working at their own company or store were divided into self-employed and employer; those having at least one employee were defined as employers, others were classified as self-employed. Participants who worked more than 18 hours per week in a company owned by another family member but who were not paid were considered unpaid family workers.

We also dichotomized several health-related conditions at baseline to consider them as potential confounders in the analysis: participants with any physical/mental disability (vs none), participants with any chronic disease (vs none), participants with poor self-rated health (vs good self-rated health), and current smokers (vs non-smoker). Self-rated health condition was originally measured using the question, “How would you rate your overall health?” The 5-point ordinal scale answer was then classified into two levels: poor (very poor, poor) and good (fair, good, excellent) in the analysis. Although current smoking status was not significantly related to depression or social capital in the present analyses (Tables 1 and 2), it was included as a potential confounder because studies strongly suggest that it is associated with both [30], [31], [32], [33], [34], [35], [36].

**Table 1:** Distribution of Study Population and Incidence of Depression by Key Covariates at Baseline (2006) in South Korea.

	Study population for new-onset depression analysis (n = 8,755)			Study population for long-term depression analysis (N = 7,939)		
	Distribution	Incidence		Distribution	Incidence	
	N (%)	N (%)	P-value*	N (%)	N (%)	P-value*
Sex			<0.001			<0.001
Male	4186 (47.8)	599 (14.3)		3756 (47.3)	160 (4.3)	
Female	4569 (52.2)	934 (20.4)		4183 (52.7)	310 (7.4)	
Age (years)			<0.001			<0.001
18 – 24	611 (7.0)	88 (14.4)		522 (6.6)	19 (3.6)	
25 – 34	1770 (20.2)	221 (12.5)		1567 (19.7)	48 (3.1)	
35 – 44	1871 (21.4)	275 (14.7)		1655 (20.8)	67 (4.0)	
45 – 55	1401 (16.0)	226 (.16.1)		1280 (16.1)	66 (5.2)	
55 – 65	1370 (15.6)	270 (19.7)		1290 (16.2)	86 (6.7)	
65+	1732 (19.8)	453 (26.2)		1625 (20.5)	184 (11.3)	
Education			<0.001			<0.001
Junior high or less	3348 (38.2)	814 (24.3)		3132 (39.5)	308 (9.8)	
High school graduate	2855 (32.6)	424 (14.9)		2569 (32.4)	106 (4.1)	
College graduate	999 (11.4)	134 (13.4)		870 (11.0)	26 (3.0)	
University graduate or more	1553 (17.7)	161 (10.4)		1368 (17.2)	30 (2.2)	
Household income			<0.001			<0.001
Less than 1Q	2188 (25.0)	582 (26.6)		2050 (25.8)	233 (11.4)	
1Q–2Q	2189 (25.0)	416 (19.0)		2002 (25.2)	123 (6.1)	
2Q–3Q	2189 (25.0)	307 (14.0)		1950 (24.6)	78 (4.0)	
3Q+	2189 (25.0)	228 (10.4)		1937 (24.4)	36 (1.9)	
Marriage			<0.001			<0.001
Currently married	6462 (73.8)	1029 (15.9)		5928 (74.7)	301 (5.1)	
Previously married	948 (10.8)	287 (30.3)		867 (10.9)	121 (14.0)	
Never married	1345 (15.4)	217 (16.1)		1144 (14.4)	48 (4.2)	
Employment status			<0.001			<0.001
Unemployed	3124 (35.7)	641 (20.5)		2844 (35.8)	229 (8.1)	
Precarious employment	1572 (18.0)	308 (19.6)		1442 (18.2)	86 (6.0)	
Unpaid family worker	616 (7.0)	131 (21.3)		579 (7.3)	44 (7.6)	
Self employed	1311 (15.0)	244 (18.6)		1217 (15.3)	74 (6.1)	
Non-precarious employment	1705 (19.5)	168 (9.9)		1500 (18.9)	33 (2.2)	
Business owner	122 (1.4)	17 (13.9)		105 (1.3)	1 (1.0)	
Student	305 (3.5)	24 (7.9)		252 (3.2)	3 (1.2)	
Current smoking			0.185			0.228
Yes	6635 (75.8)	1182 (17.8)		6068 (76.4)	370 (6.1)	
No	2120 (24.2)	351 (16.6)		1871 (23.6)	100 (5.3)	
Having any disability			<0.001			0.002
No	8289 (94.7)	1411 (17.0)		7508 (94.6)	430 (5.7)	
Yes	466 (5.3)	122 (26.2)		431 (5.4)	40 (9.3)	
Having any chronic disease			<0.001			<0.001
No	6238 (71.3)	882 (14.1)		5580 (70.3)	221 (4.0)	
Yes	2517 (28.7)	651 (25.9)		2359 (29.7)	249 (10.6)	
Self-rated health condition			<0.001			<0.001
Good	5738 (65.5)	755 (13.2)		5127 (64.6)	173 (3.4)	
Poor	3017 (34.5)	778 (25.8)		2812 (35.4)	297 (10.6)	

*P-value of the Chi-square test comparing the incidence of new-onset and long-term depression in the different groups.

**Table 2:** Individual Level Social Capital by Key Covariates at Baseline (2006) in South Korea.

	Interpersonal trust (N = 7,996)			Reciprocity (N = 8,775)			
	High	Low	P-value*	High	Intermediate	Low	P-value*
	N (%)	N (%)		N (%)	N (%)	N (%)	
Sex			0.087				<0.001
Male	2007 (52.2)	1840 (47.8)		3,054 (73)	750 (17.9)	382 (9.1)	
Female	2085 (50.3)	2064 (49.7)		3,016 (66)	955 (20.9)	598 (13.1)	
Age (years)			0.091				<0.001
18 – 24	271 (52.2)	248 (47.8)		443 (72.5)	132 (21.6)	36 (5.9)	
25 – 34	797 (50.7)	775 (49.3)		1278 (72.2)	393 (22.2)	99 (5.6)	
35 – 44	903 (53.8)	776 (46.2)		1449 (77.4)	320 (17.1)	102 (5.5)	
45 – 55	668 (51.1)	638 (48.9)		1055 (75.3)	223 (15.9)	123 (8.8)	
55 – 65	616 (48.2)	662 (51.8)		912 (66.6)	267 (19.5)	191 (13.9)	
65+	837 (51.0)	805 (49.0)		933 (53.9)	370 (21.4)	429 (24.8)	
Education			<0.001				<0.001
Junior high or less	1529 (48.7)	1608 (51.3)		2029 (60.6)	673 (20.1)	646 (19.3)	
High school graduate	1279 (49.5)	1303 (50.5)		2112 (74.0)	549 (19.2)	194 (6.8)	
College graduate	443 (50.0)	443 (50.0)		730 (73.1)	208 (20.8)	61 (6.1)	
University graduate or more	841 (60.5)	550 (39.5)		1199 (77.2)	275 (17.7)	79 (5.1)	
Household income			<0.001				<0.001
Less than 1Q	1040 (50.7)	1013 (49.3)		1340 (61.2)	426 (19.5)	422 (19.3)	
1Q–2Q	979 (49.0)	1017 (51.0)		1542 (70.4)	413 (18.9)	234 (10.7)	
2Q–3Q	961 (48.8)	1009 (51.2)		1567 (71.6)	437 (20.0)	185 (8.5)	
3Q+	1112 (56.2)	865 (43.8)		1621 (74.1)	429 (19.6)	139 (6.3)	
Marriage			0.305				<0.001
Currently married	3078 (51.6)	2883 (48.4)		4553 (70.5)	1214 (18.8)	695 (10.8)	
Previously married	429 (49.0)	446 (51.0)		525 (55.4)	209 (22.0)	214 (22.6)	
Never married	585 (50.4)	575 (49.6)		992 (73.8)	282 (21.0)	71 (5.3)	
Employment status			<0.001				<0.001
Unemployed	1339 (47.4)	1448 (52.6)		2023 (64.8)	619 (19.8)	482 (15.4)	
Precarious employment	725 (50.2)	720 (49.8)		1120 (71.2)	318 (20.2)	134 (8.5)	
Unpaid family worker	293 (50.3)	290 (49.7)		392 (63.6)	128 (20.8)	96 (15.6)	
Self employed	644 (51.6)	603 (48.4)		901 (68.7)	236 (18.0)	174 (13.3)	
Non-precarious employment	884 (57.9)	643 (42.1)		1297 (76.1)	333 (19.5)	75 (4.4)	
Business owner	67 (61.5)	42 (38.5)		98 (80.3)	17 (13.9)	7 (5.7)	
Student	140 (54.3)	118 (45.7)		239 (78.4)	54 (17.7)	12 (3.9)	
Current smoking			0.644				0.228
Yes	3105 (51.3)	2945 (48.7)		4533 (68.3)	1300 (19.6)	802 (12.1)	
No	987 (50.7)	959 (49.3)		1537 (72.5)	405 (19.1)	178 (8.4)	
Having any disability			0.195				0.002
No	3886 (51.3)	3682 (48.7)		5776 (69.7)	1619 (19.5)	894 (10.8)	
Yes	206 (48.1)	222 (51.9)		294 (63.1	86 (18.5)	86 (18.5)	
Having any chronic disease			0.001				<0.001
No	2954 (52.3)	2691 (47.7)		4564 (73.2)	1192 (19.1)	482 (7.7)	
Yes	1138 (48.4)	1213 (51.6)		1506 (59.8)	513 (20.4)	498 (19.8)	
Self-rated health condition			<0.001				<0.001
Good	2774 (53.2)	2439 (46.8)		4266 (74.3)	1055 (18.4)	417 (7.3)	
Poor	1318 (47.4)	1465 (52.6)		1804 (59.8)	650 (21.5)	563 (18.7)	

*P-value of the Chi-square test comparing the distribution of individual level social capital in the different groups.

### Data analysis

**Figure 1:**
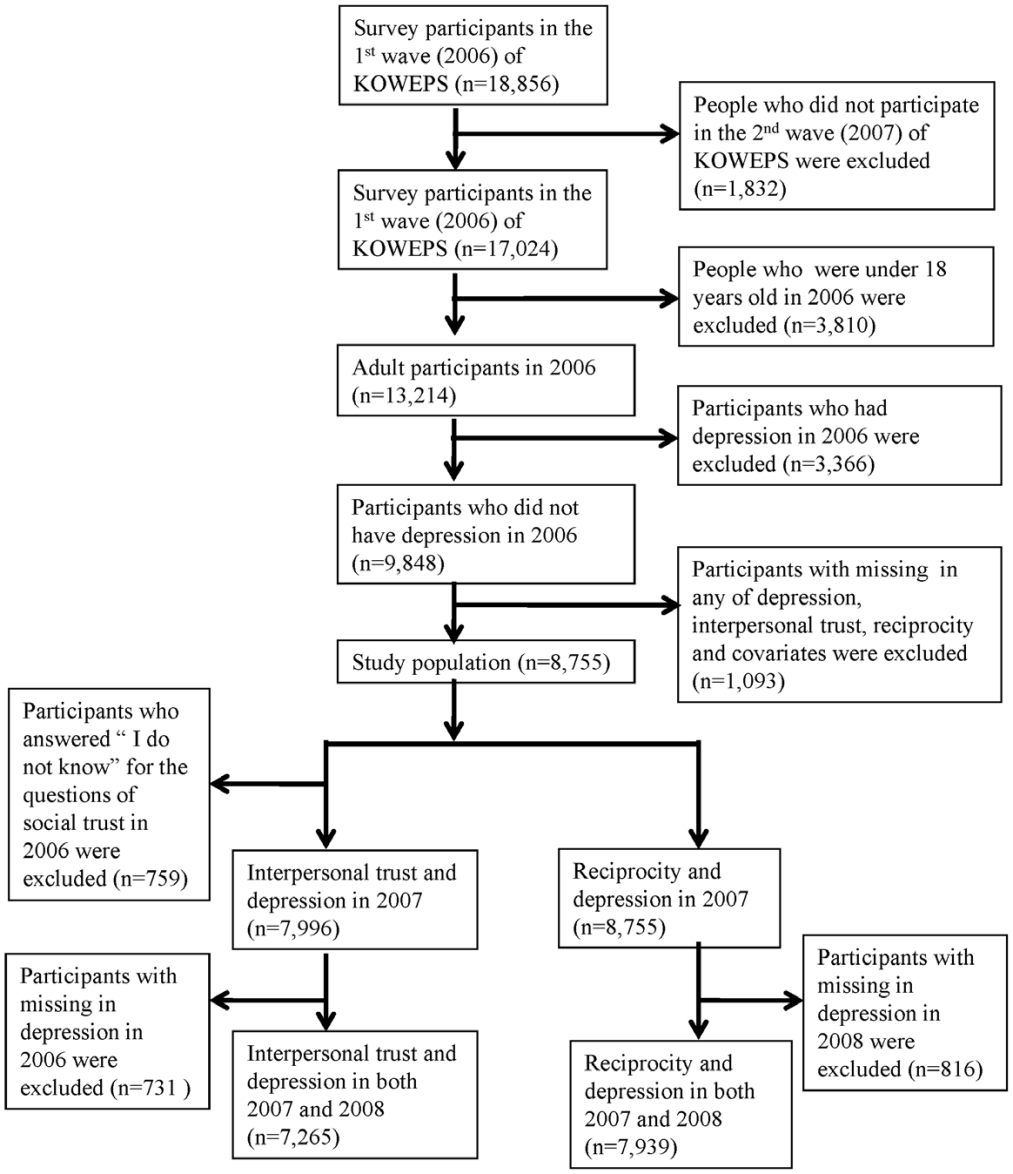
Flow Chart of Data Analyses.

The study population (hereafter, full population) includes participants who were not depressed at baseline and had information about depression in 2007 for new-onset depression and in both 2007 and in 2008 for long-term depression. Such population construction allows for examining the association between social capital and depression prospectively. When assessing the association between interpersonal trust and depression, people who answered “I do not know” were excluded from the study population. Removing participants with missing values either for social capital variables or for any potential confounder, the sample sizes were 7,996, 7,265, 8,775, and 7,939 for investigating the relationship between each of the two social capital variables and each of the new-onset and long-term depressions, respectively (Figure 1). For a sensitivity analysis we generated a smaller study population (hereafter, subpopulation) that excludes unhealthy participants with pre-existing disability, chronic disease, or poor self-rated health condition at baseline. This resulted in sample sizes of 4,645, 4,140, 5,136, and 4,569 in each of the four aforementioned association analyses (Table 3).

**Table 3:** Association Between Individual-level Social Capital and New-onset (2007) and Long-term (2007 & 2008) Depression in South Korea.

Individual-level social capital at baseline (2006)		Depression	Full population							Subpopulation^a^						
			N	Unadjusted			Fully adjusted^b^			N	Unadjusted			Fully adjusted^b^		
				OR	95% CI		OR	95% CI			OR	95% CI		OR	95% CI	
Interpersonal trust	High	2007	7996	1	Referent		1	Referent		4645	1	Referent		1	Referent	
	Low			1.28***	1.14	1.44	1.22**	1.08	1.38		1.37**	1.15	1.63	1.32**	1.10	1.57
	High	2007 & 2008	7265	1	Referent		1	Referent		4140	1	Referent		1	Referent	
	Low			1.33*	1.10	1.62	1.23*	1.03	1.50		1.59**	1.13	2.22	1.47*	1.05	2.08
Reciprocity	High	2007	8755	1	Referent		1	Referent		5136	1	Referent		1	Referent	
	Intermediate			1.32***	1.15	1.51	1.20*	1.04	1.38		1.09	0.89	1.35	1.04	0.84	1.29
	Low			1.89***	1.61	2.21	1.32**	1.11	1.56		1.36*	1.01	1.83	1.20	0.88	1.63
	High	2007 & 2008	7939	1	Referent		1	Referent		4569	1	Referent		1	Referent	
	Intermediate			1.34*	1.06	1.69	1.15	0.91	1.47		0.83	0.53	1.30	0.77	0.49	1.23
	Low			1.95***	1.51	2.51	1.11	0.84	1.45		1.44	0.83	2.50	1.17	0.66	2.06

*: *P*<0.05;

**: *P*<0.01;

***: *P*<0.001

a : The population restricted to healthy people includes the participants who did not have disability, chronic disease, or poor self-rated health condition at baseline (2006).

b : Adjusted for gender, age, education level, income level, marital status, employment status, smoking status, disability, chronic disease, and self-rated health condition at baseline(2006).

A logistic regression model was applied to investigate the association between individual-level social capital and depression. The Generalized Estimating Equation method was adopted to estimate the regression parameters accounting for correlations among individuals within a household. We examined the associations between each of the two social capital indicators and each of the new-onset and long-term depressions separately after adjusting for potential confounders. After finding a significant relationship between social capital and depression in the full study population, we checked the association in the subpopulation after excluding unhealthy participants with disability or chronic disease or poor-self rated health. Participants with high interpersonal trust (vs low) and with high reciprocity (vs intermediate and low) were used as reference groups. The associations were summarized as the estimated odds ratios with 95% confidence intervals. Two-sided p-values are presented in the tables. All analyses were performed using STATA/SE version 11.0 (StataCorp, College Station, TX).

### Ethics

The KOWEPS is the publicly released dataset that is available at the website of the Korea Welfare Panel Study (http://koweps.re.kr/). Informed consent was not required to use this dataset. This research received IRB exemption from the Office of Human Research Administration at the Harvard School of Public Health.

## Results

Table 1 shows the distribution of the study population and the incidence of depression across different levels of each confounder. The overall incidence was 17.5% (1,533 out of 8,755 participants) for new-onset depression and 5.9% (470 out of 7,939 participants) for long-term depression. The incidence of depression was higher for females and the elderly. The same was true for participants with lower education or income levels. Previously married people were more likely to develop depression compared to those never or currently married. Unemployed, precariously employed, self-employed, and unwaged workers all exhibited higher incidences of depression compared to non-precarious employees and students. Depression appeared to be more common for people having disability, chronic disease, or poor self-rated health condition.

Table 2 summarizes the distribution of social capital by each confounding variable. Lower levels of reciprocity were found for participants who were female, older, lower-educated, or in a lower income level compared to their counterparts. Previously married participants exhibited a lower level of reciprocity than those never or currently married. Reciprocity was also lower for the unemployed, self-employed, precariously-employed, and unwaged family workers than non-precarious workers, business owners, and students. Participants having disability, chronic disease, or poor health were more likely to display lower reciprocity.

Interpersonal trust was significantly associated with new-onset depression as well as long-term depression in both populations after adjusting for all confounders (Table 3). In the full population, participants with low interpersonal trust had 23% higher odds of developing long-term depression (OR: 1.23; 95% CI: 1.03, 1.50). In the subpopulation, after excluding unhealthy participants, the association became stronger, and those participants with low interpersonal trust had 47% higher odds of developing long-term depression (OR: 1.47; 95% CI: 1.05, 2.08).

Reciprocity was significantly related with new-onset depression but not with long-term depression in the full population (Table 3). There were 20% higher odds of developing new-onset depression for participants with intermediate reciprocity (OR: 1.20; 95% CI: 1.04, 1.38) and 32% higher odds for participants with low reciprocity (OR: 1.32; 95% CI: 1.11, 1.56). However, the association was attenuated and non-significant for long-term depression in the full population and for both new-onset and long-term depression in the subpopulation after we excluded unhealthy people.

## Discussion

Our findings consistently suggest that low interpersonal trust at an individual level is an independent predictor of new-onset depression. Using nationally representative data from South Korea, the odds for new-onset and long-term depression were significantly (22 – 47%) higher for lower-level interpersonal trust compared to participants with higher-level interpersonal trust after adjusting for potential confounders. For interpersonal trust, the relationship remained significant and became stronger in the subpopulation analyses after excluding participants with preexisting unhealthy conditions. Our results are consistent with previous findings showing that individual-level interpersonal distrust or hostility is independently associated with depression [6], [37], [38].

In contrast, we could not find significant associations between low and intermediate levels of reciprocity and long-term depression in the fully adjusted models. And associations between low and intermediate levels of reciprocity and new-onset depression, although significant in the full-population analyses, were attenuated and became non-significant in the subpopulation analyses restricted to healthy people, implying that the impact in the full population was mediated by other health-related conditions. These different impacts on depression between interpersonal trust and reciprocity are consistent with results of a previous study, which found a strong association between individual-level interpersonal trust and depression but no association for individual-level reciprocity in fully adjusted model [6]. This difference might result from measuring different aspects of social capital. Torche and Valenzuela [39] suggested that interpersonal trust measures people's perceptions about their relationships within a bounded community, and thus tends to reflect relationships between those with similar backgrounds in terms of residential area, education level, economic status, etc. In contrast, reciprocity is more likely to measure people's perceptions about strangers, and thus encompasses relationships across different socio-economic backgrounds.

Our results indicate that, for associations of interpersonal trust and reciprocity with depression, future research should consider the socio-political contexts of South Korea. South Korea has recently experienced rapid social change: economic development resulted in disorganization of traditional communities in rural areas and centralization of socio-cultural human resources in metropolitan areas. The culture has also been strongly influenced by Confucianism. These unique socio-political contexts introduce variables absent from those Western countries where most prior social capital studies were performed. In addition, studies in political science also suggest that the effect of social capital in South Korea could differ from that in Western countries [40], [41], [42].

The underlying mechanisms linking individual-level social capital and depression are not well established [17], but previous studies suggest that they may resemble those proposed for the neighborhood level [4], [5]. Two models have been suggested to explain the protective effect of high interpersonal trust on mental health: the main effect model and the stress-buffer model [6], [43]. The main effect model suggests that living in a trusted neighborhood can provide an individual with a sense of belonging within the community. The stress-buffer model hypothesizes that neighborhood interpersonal trust can provide emotional support to help people deal with daily stress. Adapting these models to our understanding of social capital and depression, however, requires further evaluation, specifically accommodating the socio-political context of South Korea.

One of the major strengths of this study is the large, nationally representative sample of the South Korean population. Additionally, we found strong associations between interpersonal trust and depression after adjusting for potential confounders; this relationship was consistent even in the subpopulation analyses where participants with poor health conditions were excluded. Third, depression was measured using a standardized method, CES-D, which has been validated [27] and commonly employed in previous research in South Korea [44], [45], [46]. Finally, to our knowledge, this is the first study to examine the association between social capital and mental health outcome in East-Asian countries using a prospective study design.

The present analyses have at least three limitations. First, although we performed multiple adjustments, potential confounders may have gone unmeasured. Specifically, we did not have information about previous medical history of mental or physical disease; these represent potential confounders because they can be associated with social capital and also can be risk factors of new-onset and long-term depression. However, because strong associations were detected in the subpopulation analyses after excluding unhealthy participants, our results are expected to be relatively robust against these unadjusted confounders.

Second, the present analyses assessed only individual-level cognitive components of social capital. Previous studies suggest there are three types of social capital, specifically, bonding, bridging, and linking social capital [7], [47], [48], and each type of social capital can be composed of structural components (for example, access to public goods and services) and cognitive components (for example, interpersonal trust and norm of reciprocity) [48], [49]. The present analyses used the cognitive components of bonding social capital, which have been most commonly adopted in previous studies and showed a strong association with mental health outcome [17]. Future research is required to assess the health impact of different levels of social capital in South Korea, particularly including a structural component as a community-level resource.

Finally, no previous studies have checked the validity of social capital measurement in South Korea, and the social environment of South Korea may have introduced an additional issue. For example, measuring the density of membership in civic associations might not be appropriate in South Korea because most Koreans forego formal associations for small informal groups [42]. Moreover, previous studies in political science showed that social capital has a relatively minor or little effect on political trust/activism/participation in South Korea [40], [41], [42], which differs from prior findings in westernized countries. Thus, different measures of social capital may be more valid in South Korea.

### Conclusions

This study, using nationally representative data for the South Korean population, showed that individual-level interpersonal trust is a strong predictor of both new-onset and long-term depression after adjustment of confounders. This association was found to be stronger in the subpopulation analyses after the exclusion of the participants with pre-existing poor health condition. In contrast, an association between individual-level reciprocity and new-onset depression was attenuated and became non-significant after adjustment for confounders in the analyses restricted to healthy populations. Further study is required to examine the validity of the instrument to measure social capital and to reveal a mechanism of how different types of individual social capital (individual interpersonal trust and reciprocity) could be related to mental health in South Korea.
